# Health Tract, No. 42

**Published:** 1865

**Authors:** 


### HEALTH TRACT, No. 42.

---

### HEALTH'S THREE ESSENTIALS.

All who are now in health can keep well, and three out of four of those suffering from the common transient ailments of life can be perfectly cured by giving a steady, judicious attention to the three following rules:

### RULE FIRST.

Never eat between meals, nor take anything for supper but a single piece of cold bread and butter and a glass of water, or one cup of any kind of hot drink.

### RULE SECOND.

Secure one regular, free, and full daily action of the bowels every morning after breakfast by the use of your ordinary food (see Health Tract No. 22); and to this end do not leave your home under any pretense, for a single moment, until there is an inclination to stool; then, as you value a long and healthful life, do not defer the call for a single second of time, for anything short of a fire or a fit; rather cherish the inclination. If it does not come within half an hour of the regular time, solicit nature. If unsuccessful, do not eat an atom of anything until the passage is secured, or at least until next morning. Meanwhile drink as much cold water or hot tea as you desire, and keep exercising (tenfold better if in the open air) to the extent of sustaining a scarcely perceptible perspiration for the greater part of the day; for it must strike you that if food is steadily passed into the mouth, and there is no corresponding outlet, harm is absolutely inevitable. If, during the second day, the bowels do not move, call in a regularly educated physician.

### RULE THIRD.

Cool off very slowly after all forms of exercise; the neglect of this lights up the fires of three fourths of all the diseases which afflict humanity. Cool off slowly by putting on more clothing than while exercising instead of laying aside some, even a hat or a bonnet; go to a closed room rather than sit or stand out of doors; sit by a good fire rather than an open window; at all events keep in motion in such a way as to allow the perspiration, or any extra warmth, to disappear very gradually indeed.

If a fourth rule were added, it should be to keep one end of the body, the feet, always dry and warm (see Health Tract No. 21), and the other, the head, cool and clean, by spending two minutes in midwinter, and five or more in midsummer, in washing, with ordinary cold water, the scalp, if the hair is short, the ears, neck, throat, arm-pits, upper part of chest and arms; rub dry briskly, dress quickly, and go to breakfast.

These same observances (the first three) will incalculably mitigate every disease to which mortal man is subject — will moderate every pain and will soothe every sigh; and a pity is it beyond expression, that every human creature does not know and habitually practice them.

